# Lymphoepithelial cyst of the pancreas: can common imaging features help to avoid resection?

**DOI:** 10.1007/s00423-023-02777-9

**Published:** 2023-02-11

**Authors:** Ekaterina Khristenko, Elena Esteban Garcia, Matthias M. Gaida, Thilo Hackert, Philipp Mayer, Hans-Ulrich Kauczor, Miriam Klauss

**Affiliations:** 1 Department of Diagnostical and Interventional Radiology, Heidelberg University Hospital, Im Neuenheimer Feld 420, 69120 Heidelberg, Germany; 2 Department of Radiology, Sofia General University Hospital, Av. Intendente Jorge Palacois, 1, 30003 ReinaMurcia, Spain; 3 Department of Pathology, Johannes Gutenberg University, Saarstrasse 21, 55122 Mainz, Germany; 4 Department of General, Visceral, and Transplantation Surgery, Heidelberg University Hospital, Im Neuenheimer Feld 420, 69120 Heidelberg, Germany

**Keywords:** LEC, Pancreatic cysts, Imaging features, Pancreatic surgery, Prognosis

## Abstract

**Background:**

Differentiation of cystic pancreatic neoplasms remains a challenging task for radiologists regarding the main aim of identifying malignant and premalignant lesions.

**Purpose:**

The study aimed to compare the radiological features of lymphoepithelial cysts (LEC) with other cystic pancreatic lesions, which could help to differentiate them in order to avoid unnecessary resection.

**Material and methods:**

We retrospectively reviewed 10 cases of resected and histopathologically confirmed LECs during a 12-year period with available imaging studies; 20 patients with mucinous cystic neoplasms (MCN), 20 patients with branch-duct intraductal papillary mucinous neoplasms (BD-IPMN), and 20 patients with serous cystic neoplasms (SCN) were selected to serve as control groups. Imaging findings as well as clinical data were analyzed.

**Results:**

The following imaging morphology of LEC was identified: simple cystic appearance (20%) and mixed cystic-solid appearance (80%) with either a diffuse subsolid component (30%) or mural nodule(s) (50%). All lesions revealed exophytic location with a strong male predominance (9:1). MCNs occurred exclusively in middle-aged women, IPMN in both sexes showed slight male predominance (13:7), and SCN showed female predominance (5:15). Median patient age in LEC (48.5, IQR 47–54.5) was significantly younger compared to IPMN (*p* < 0.001) and SCN (*p* = 0.02). Unenhanced CT attenuation of LEC was higher than MCNs (*p* = 0.025) and IPMNs (*p* = 0.021), showing no significant difference to SCN (*p* = 0.343).

**Conclusion:**

The present study provides key radiological features of LEC for the differentiation from other cystic pancreatic lesions such as increased CT attenuation in the unenhanced phase, absence of a connection to the main pancreatic duct (MPD), and exophytic location. In addition to these imaging features, clinical data, such as male predominance in LEC, must be considered for the differentiation of cystic pancreatic lesions.

## Introduction

Cystic pancreatic lesions are divided into neoplastic and non-neoplastic lesions, with the bigger part being neoplastic lesions. Most commonly, neoplastic lesions include intraductal papillary mucinous neoplasms (IPMN), mucinous cystic neoplasms, serous cystic neoplasms (SCN), and solid pseudopapillary tumor, which account for approximately 70% of all cystic pancreatic lesions. Non-neoplastic lesions are divided into epithelial and nonepithelial lesions, with the most common epithelial lesions being congenital cysts, alimentary duplication cysts, endometrial cysts, and lymphoepithelial cysts (LEC), and the most common nonepithelial lesions being pseudocysts or walled-off necrosis [1].

LECs of the pancreas are rare benign cystic lesions. They were first described in 1985 by Luchtrat [2], and the majority of available data on the radiological features of LECs are case reports. Over the past 30 years, since the first description, app. 100 cases were reported [3]. As they are rare, their clinical and pathologic features have not yet been fully characterized, especially regarding the differentiation from other cystic lesions [4, 5]. In the clinicopathologic analysis of 12 patients, LECs were reported to constitute approximately 0.5% of pancreatic cysts and were seen in middle-aged patients and predominantly, but not exclusively in men (M/F = 4/1) [6]. Recent reviews documenting the demographic features of LECs indicate a strong male preponderance as well [7].

LECs are true pancreatic cysts that are lined by squamous, non-dysplastic epithelium and surrounded by mature lymphoid tissue [8]. Etiological theories include origination from squamous metaplasia of pancreatic ducts, derivation from epithelial remnants in lymph nodes as well as displacement of ultimo-branchial remnants that go on to fuse with the pancreas during embryogenesis. There is also a possibility that LECs are a distinct form of teratoma [6].

Because the imaging features of the LECs vary and sometimes are very similar to other pancreatic lesions, it is difficult to differentiate LECs from other pancreatic lesions. However, correct differentiation and classification are crucial for clinical decision-making and treatment planning. LECs are benign and do not possess a malignant potential, and thus, accurate identification of these lesions is important to avoid unnecessary intervention [8]. A correct preoperative diagnosis of an LEC could help prevent surgical interventions, although today, fine needle aspiration is the only tool that can achieve a definitive diagnosis without resection [3, 9–11].

Both MCNs and IPMNs are characterized by a neoplastic mucin-producing epithelial cell lining with the potential for malignant transformation. Since the rare data about imaging features of LEC that are available from case series show an overlapping with MCNs, SCNs, and IPMNs, differentiation of LEC from these potentially malignant pancreatic lesions remains difficult and is challenging from the radiological point. The present study aimed to compare LECs with these cystic lesions of the pancreas and to identify common radiological features which could help to differentiate them from other cystic neoplasms in order to avoid unnecessary resections.

## Methods

### Ethics approval and consent

This study was performed according to the Declaration of Helsinki. Our study was approved by the Institutional Review Board (IRB) of Heidelberg University Hospital (Number S 011/15). Due to its retrospective design, the informed consent for the patients was waived by the IRB.

### Patient selection and data collection

We reviewed 10 cases of resected and histopathological confirmed LECs of the pancreas in our institution (Heidelberg University Hospital) during the last 12 years with available clinical and imaging data; 20 patients with MCNs, 20 patients with SCNs, and 20 patients with branch-duct IPMNs, who underwent resection during the same time period, were selected as matched-pair out of the institutional study collective and served as control groups. Therefore, the study included 70 patients with cystic pancreatic lesions, among them 10 patients with LECs, 20 patients with MCNs, 20 patients with SCNs, and 20 patients with branch-duct IPMNs. The patients with IPMN were selected from our prospectively followed institutional study collective of 1271 patients with histologically confirmed BD-IPMNs. We excluded the malignant IPMNs with invasive components as they generally show a solid lesion by the time of diagnosis, as our aim was to compare the average radiological appearance of LEC with the average appearance of BD-IPMN by the time of first diagnosis.

Patients with pancreatic pseudocysts were not included in the study as these lesions are benign and could be better differentiated with clinical data, such as episodes of acute pancreatitis. Data such as symptoms, laboratory tests, histopathological findings, follow-up, and imaging findings were retrospectively analyzed for all patients.

### Diagnostic imaging

Due to the retrospective design of the present study, imaging was not standardized. In the LEC group, three patients underwent both preoperative CT and MRI, four underwent MRI, and three underwent CT only.

CT examinations were performed on scanners with either 16 or 64 rows (Siemens Healthcare, Forchheim, Germany; Philips Healthcare, Best, Netherlands; GE Healthcare, New York, USA). Soft tissue kernels were used for multiplanar reconstruction. All CTs included unenhanced images and two-phase contrast-enhanced images after intravenous injection of nonionic contrast medium. Coronal reconstructions in the venous (*n* = 6) and both arterial and venous (*n* = 4) phases were available.

MRI with magnetic resonance cholangiopancreatography (MRCP) was available for 7 patients. MR imaging was performed on scanners from the same companies as the CT scanners with field strengths ranging from 1 to 1.5 Tesla. All MRI studies included the following imaging sequences: T2-weighted axial images without fat suppression (fs) and coronal T2-weighted images with or without fat suppression (fs). T1-weighted images before and after contrast injection were obtained with dynamic protocol after the injection of extracellular contrast agent as a bolus injection of 0.2 ml/kg gadolinium chelate. The arterial phase was defined as full enhancement of hepatic arteries and absence of enhancement of hepatic veins, the portal venous phase was defined as full enhancement of portal veins and antegrade enhancement of hepatic veins, and the late phase was defined as similar enhancement of portal veins and hepatic veins and enhancement of liver parenchyma to a lesser degree than in portal venous phase. Diffusion-weighted images were available for 4 patients and included *b*-values of 0 s/mm^2^, 50 s/mm^2^, and 800 s/mm^2^.

For the control groups, imaging studies were performed with a standard imaging protocol used in our hospital. For all of them, both CT (unenhanced and contrast-enhanced imaging) and MR imaging were available. CT examinations were performed on a scanner with 64 rows (Siemens Healthcare, Forchheim, Germany). MR imaging was performed on scanners with a field strength of 1.5 Tesla (Siemens Healthcare, Forchheim, Germany).

### Radiological evaluation

Two radiologists (EK with 10 years of experience in abdominal imaging and EEG with 2.5 years of experience), blinded to the histopathological reports, analyzed the images independently, using a Picture Archiving and Communication System (PACS) workstation. Imaging analysis included the following parameters: location (head, body, tail), lesion appearance (cystic, subsolid), septation (present, absent), enhancing mural nodules (present, absent), and dilatation of main pancreatic duct (MPD) > 3 mm and common bile duct (CBD) > 8 mm upstream of the lesion (present, absent; for lesions without proximity to the MPD or CBD and for lesions located at the tip of the pancreatic tail the latter two parameters were classified as not applicable). The size of lesions (mm) and attenuation values (Hounsfield units, HU) on unenhanced images as well as in the arterial and venous phases were measured. To assess the attenuation on unenhanced, arterial, and venous CT scans, HU were measured using circular regions of interest (ROI) located in the most homogeneous part of the lesion. Afterward, discrepancies in image interpretation were resolved by consensus between the two radiologists.

The following parameters were assessed for CT images only: calcification (present, absent) and CT density on unenhanced phase (hypodense, isodense, hyperdense).

The following parameters were assessed for MR images only: signal intensity (hypointense, isointense, hyperintense) and signal uniformity (homogeneous, heterogeneous) of the lesion on unenhanced T1- and T2-weighted imaging.

### Statistical analysis

Data management was carried out by SAS software release 9.4 (SAS Institute, Cary, North Carolina, USA), and statistical analysis was made using IMB SPSS software, version 24 (IBM Corp.). Data are presented as median with interquartile range (IQR). Mann–Whitney U test was performed to compare continuous parameters between groups. For categorical parameters, absolute numbers are shown. Two-sided *p*-values were computed; the differences were considered statistically significant at a *P*-value of 0.05 or less.

## Results

### Surgical procedure and histopathology

The patients were considered symptomatic if they had a left upper quadrant (LUQ) or epigastric pain, post-prandial complaints (i.e., cramps), and did not have other specific causes for these symptoms. Clinical symptoms and the radiological preoperative diagnosis based on the imaging morphology of the lesion served as the main indications for surgical treatment.

LEC patients underwent cyst resection or left (distal) pancreatic resection with or without splenectomy. After surgery, 60% of the patients had minor local complications: Four had postoperative fluid collections to be drained, one had a pancreatic fistula, and one developed a pseudocyst.

Histopathological reports confirmed LECs of the pancreas in all 10 patients without evidence of malignant transformation. In the MCN group, there were 14 low-grade MCNs, 4 high-grade MCNs, and 2 MCNs with associated invasive carcinoma. In the IPMN group, there were 18 BD-IPMN and 2 mixed-type IPMN, with 14 low-grade IPMN and 6 IPMN with carcinoma in situ. In the SCN group, there were 18 SCN of the microcystic type and 2 SCN with of the oligocystic type.

### Patient characteristics/demographics

LECs were more common in middle-aged males (nine males, one female), ranging from 43 to 63 years (median 48.5, IQR 47–54.5). A summary of patient demographics, initial LEC findings, and surgical procedures is presented in Table 1.

**Table 1:** Summary of patient demographics, initial LEC findings, and surgical procedures

	Gender	Age	Modality	Location	Size (cm)	Size of mural nodule (cm)	Preoperative diagnosis	Surgical procedure
Pat. 1	Male	59	CT	Tail	2.7		Side branch IPMN	Cyst enucleation with lymphadenectomy
Pat. 2	Male	53	CT	Tail	5.0	1.1	MCN	Left resection with splenectomy
Pat. 3	Male	47	CT	Tail	4.4		SCN	Left resection with splenectomy
Pat. 4	Male	63	MRI	Body	5.8		MCN	Left resection with splenectomy
Pat. 5	Male	49	MRI	Tail	4.4	2.4	MCN	Left resection with splenectomy
Pat. 6	Male	44	MRI	Tail	3.6	0.8	Side branch IPMN	Cyst enucleation
Pat.7	Male	55	MRI	Body	3.6		Side branch IPMN	Left resection with splenectomy
Pat. 8	Male	48	CT/MRI	Body	4.5		SCN	Cyst enucleation
Pat. 9	Male	44	CT/MRI	Tail	5.3	0.7	Side branch IPMN	Left resection
Pat. 10	Female	47	CT/MRI	Tail	22	5.6	MCN	Left resection with splenectomy

*LEC*, lymphoepithelial cyst; *CT*, computed tomography; *IPMN*, intraductal papillary mucinous neoplasm; *MCN*, mucinous cystic neoplasm; *SCN*, serous cystic neoplasm; *MRI*, magnetic resonance imaging

In contrast, MCNs were detected exclusively in middle-aged women with the age range of 37 to 67 years (median 51, IQR 41–57). There was no statistically significant difference in age between the LEC group and the MCN group (*p* = 0.983).

Branch-duct IPMNs were found in both sexes showing a slight male predominance (13 male and 7 female) with an age range of 55 to 78 years. The median patient age was higher compared to the other two groups (median 71, IQR 60–69); the difference was statistically significant (LEC compared to IPMN *p* < 0.001 and MCN compared to IPMN *p* = 0.005).

SCNs were found in 15 female patients and 5 male patients showing female predominance. The age ranged from 34 to 82 years (median 62, IQR 58–71); the difference between the age compared to LEC was statistically significant (*p* = 0.02).

### Imaging findings

All patients with LEC presented with a well-defined exophytic lesion within the tail (70%) or body (30%) of the pancreatic parenchyma without communication with the main pancreatic duct. The median size was 4.5 cm (IQR 3.8–5.4). There were no calcifications within the pancreatic parenchyma or within the cystic lesions. On unenhanced CT scans nine, LECs had high attenuating fluid or material with an unenhanced CT attenuation median value of 22 HU (IQR 19–27).

MCN lesions had a solitary/macrocystic appearance in the distal pancreas with a median size of 4.4 cm (IQR 3.6–8.6). In all cases, the pancreatic tail was involved, with 5 out of 20 occupying both the body and the tail of the pancreas. There was also no connection to the pancreatic duct.

In 13 out of 20 BD-IPMN patients, the head of the pancreas was affected (one patient with the involvement of the entire pancreas and one patient with involvement of the pancreatic head and tail), and seven were found in the pancreatic tail. The median size was significantly smaller than in the previously mentioned two groups with a median size of 2.3 cm (*p* < 0.001). The key differential feature was the finding of communication to the main pancreatic duct, which was easily confirmed using MRCP images (when available).

SCN had polycystic morphology in 18 cases and oligocystic morphology in 2 cases; 10 were situated in the pancreatic head, 2 in the pancreatic body, 7 in the pancreatic tail, and one in the entire pancreas. The median size was 4.1 cm (IQR 3.4–5.6); there was no difference when compared to LEC (*p* = 0.533). SCN also showed increased HU values in the unenhanced phase (median HU 19, IQR 18–20); there was a difference in LEC by this parameter (*p* = 0.343).

When comparing LEC with other cystic lesions, there was no statistically significant difference in size between LEC and MCN (*p* = 0.912). In contrast, BD-IPMNs were significantly smaller than LEC (*p* < 0.001). Unenhanced CT attenuation of LECs was significantly higher than both MCNs (*p* = 0.025) and IPMNs (*p* = 0.021). A summary of the comparison between the four groups is presented in Table 2.

**Table 2:** Results of the comparison between LEC, MCN, BD-IPMN, and SCN

	LEC	MCN	BD-IPMN	SCN
Number of patients	10	20	20	20
Median age (years)	48.5	51 (*p* = 0.983)	71 (*p* < 0.001)	62 (*p* = 0.02)
Gender (m:f)	9:1	1:19	13:7	5:15
Size (cm)	4.5	4.4 (*p* = 0.912)	2.3 (*p* < 0.001)	4.1 (*p* = 0.533)
Median attenuation values in the native phase, HU	22 (16 to 34 HU)	13 (10 to 21 HU) (*p* = 0.025)	12 (8 to 20 HU) (*p* = 0.021)	19 (12 to 22 HU) (*p* = 0.343)
Mural nodules (*n*)	50% (5/10)	1	0	30% (6/20)
Duct dilation 3-5 mm (*n*)	0 (< 3 mm)	0 (< 3 mm)	50% (10/20), mean 4 mm	1 (> 5 mm)

*LEC*, lymphoepithelial cyst; *MCN*, mucinous cystic neoplasm; *BD-IPMN*, branch-duct intraductal papillary mucinous neoplasm; *SCN*, serous cystic neoplasm; *HU*, Hounsfield units

**Fig. 1:**
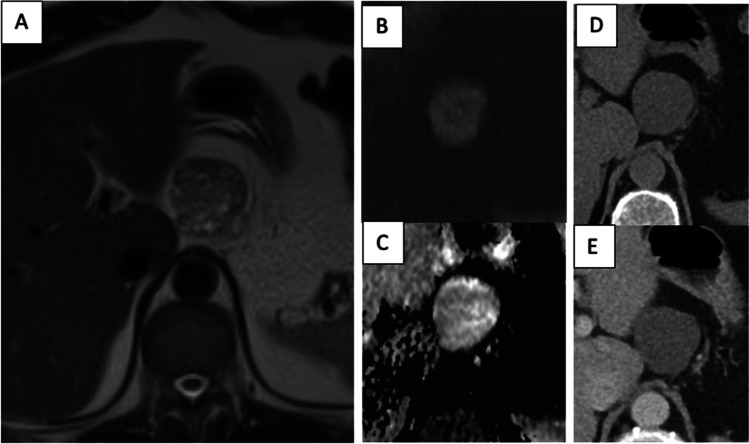
A 48-year-old patient with a 4,5 cm size lymphoepithelial cyst (LEC) in the pancreatic body with an exophytic location. Axial T2-weighted MR image (**a**) shows diffuse subsolid morphology of the lesion. Axial DWI MR image (**b**) and axial ADC MR image (**c**) show no restricted diffusion. Axial CT image in native phase (**d**) shows an isodense CT attenuation of the lesion. Axial CT image in venous phase (**e**) shows no significant enhancement of the lesion

**Fig. 2:**
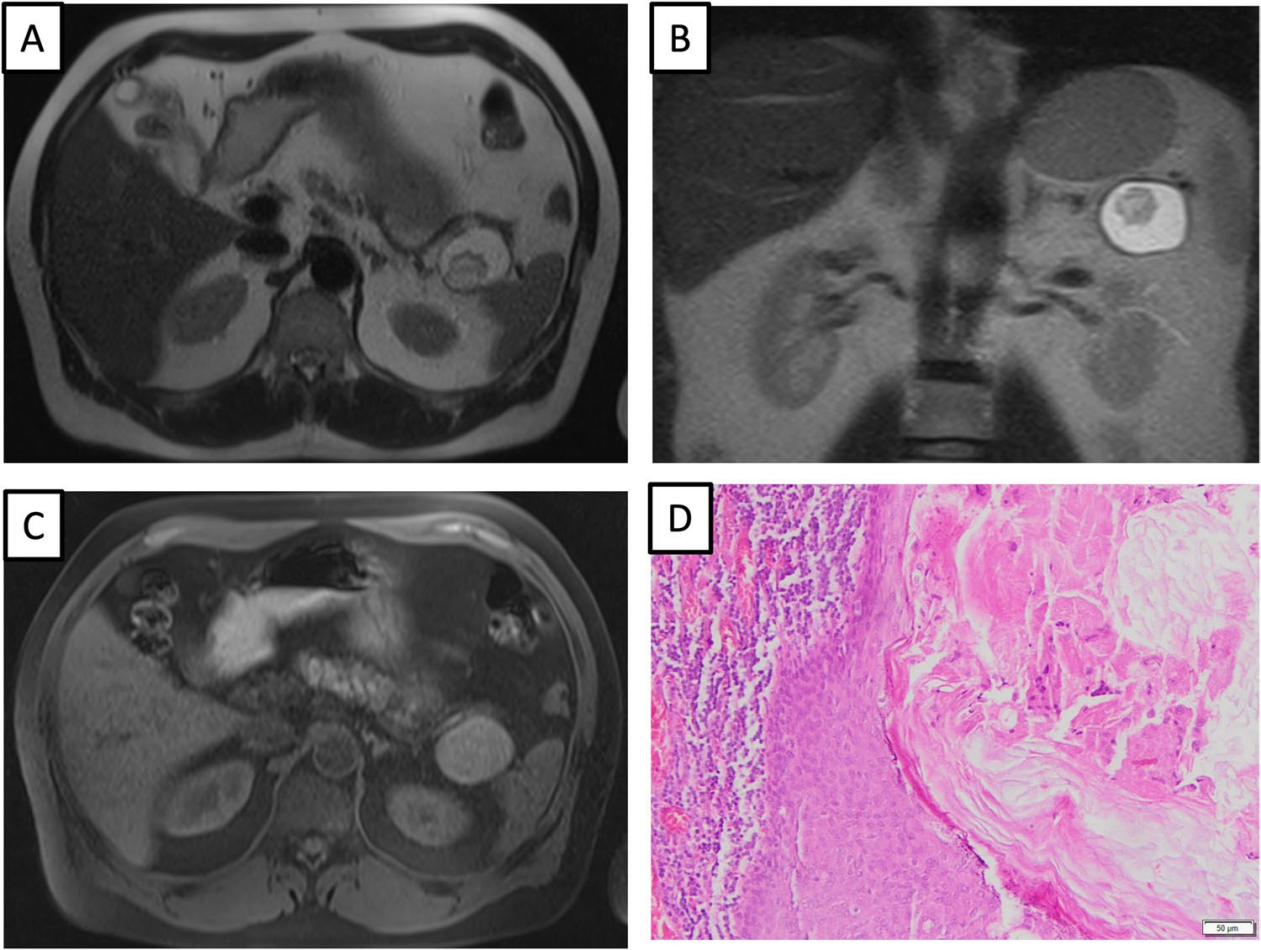
A 49-year-old male patient with lymphoepithelial cyst (LEC) in the pancreatic tail with a 4,4 cm size. Axial T2-weighted MR image without fat suppression (**a**) shows a cystic pancreatic lesion in the tail of the pancreas with a mural nodule in the dorsal part, nodule size 2,4 cm. The same lesion was demonstrated in the coronal T2-weighted MR image (**b**). Axial T1-weighted with fat suppression (**c**) shows the T1-hyperintensity of the lesion. Pathological correlation of LEC (**d**). View with high magnification shows typical squamous epithelium with keratinization and underlying “band-like” lymphocyte aggregations. No epithelial dysplasia

**Fig. 3:**
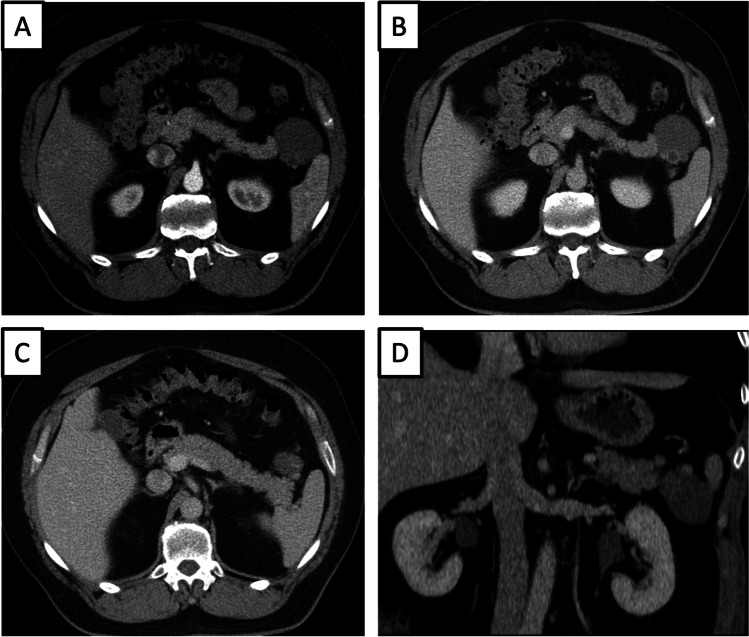
A 53-year-old male patient with lymphoepithelial cyst (LEC) in the distal part of the pancreatic tail with a mural nodule, size of the lesion 5,4 cm, size of the mural nodule 1,1 cm. Axial CT image in the arterial and venous phase (**a** and **b**) shows a cystic lesion in the distal pancreatic tail with a solid component. Axial CT image in the venous phase on a different level (**c**) demonstrates the solid component more clearly (Hounsfield units (HU) values of the solid component 38 HU in the native phase, 103 HU in the arterial phase and 90 HU in the venous phase). Multiplanar reconstruction in venous phase as correlation (**d**); the solid component is located in the cranial part

Taking the imaging features together, the following imaging morphology of LECs was defined. In 20% of cases, LECs presented as simple cystic lesions; 80% of LECs were mixed solid**-**cystic lesions with varying appearances: The first group (30%) had a diffuse subsolid component (Fig. 1), and the second group (50%) had mural nodules (Fig. 2 and Fig. 3).

The subsolid component was defined as a homogeneous or heterogeneous area of higher attenuation than simple fluid on non-contrast CT, with unchanged attenuation on the arterial/venous phase. The median size of the mural nodule was 1.1 cm, ranging from 0.8 to 5.6 cm (IQR 8–24). Four LECs were unilocular, six multilocular (≥ 4), and five had septations. Both septations and solid components showed contrast enhancement in the multiphase studies. One LEC had areas of a sponge-like appearance due to septations with some degree of enhancement, similar to those in serous cystic neoplasms.

None of the lesions caused pancreatic duct or bile duct dilation nor atrophy of the pancreatic parenchyma. This feature was useful in differentiating LECs from IPMNs, with IPMNs showing a slight dilatation of MPD in 50% of cases with a mean diameter of 4 mm (range 3 to 6 mm).

**Fig. 4:**
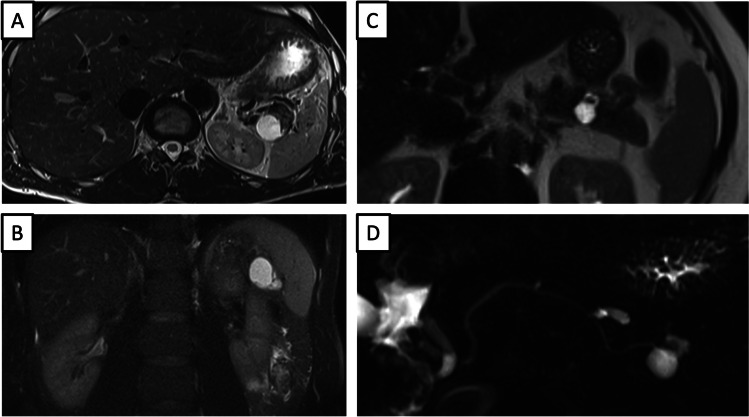
Comparison of lymphoepithelial cyst (LEC) and branch-duct intraductal papillary mucinous neoplasm (BD-IPMN). (**a**, **b**) A 44-year-old male patient with a cystic lesion within the pancreatic tail. Axial T2-weighted MR image without fat suppression (**a**) shows LEC in the distal part of the pancreatic tail with a similar image to a BD-IPMN. Coronal T2-weighted MR image with fat suppression (**b**) demonstrates the septations of the lesion. (**c**, **d**) A 48-year-old male patient with BD-IPMN in the pancreatic tail. Axial T2-weighted MR image without fat suppression (**c**) shows a side branch IPMN in the pancreatic tail. Coronal MRCP MR image (**d**) shows the communication of the lesion to the main pancreatic duct, which was the key differential feature

Overlapping imaging features of LEC and branch-duct IPMN are shown in Fig. 4. The most important differential diagnosis in the case of a small cystic lesion in the pancreatic tail is IPMN due to the morphology of the lesion. Nevertheless, there was no clear connection with the main pancreatic duct on the MRCP images, so the diagnosis was not certain.

**Fig. 5:**
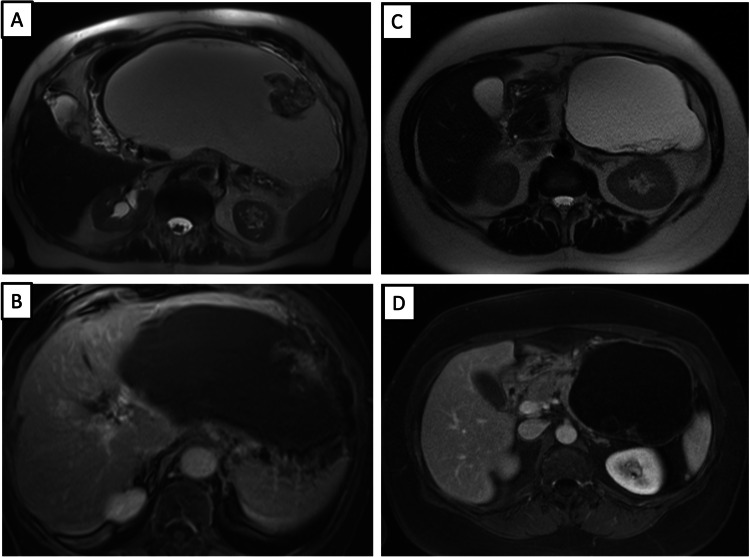
Comparison of lymphoepithelial cyst (LEC) and mucinous cystic neoplasm (MCN). (**a**, **b**) A 47-year-old female patient with huge LEC in the pancreatic body and tail, lesion size 22 cm. Axial T2-weighted MR image without fat suppression (**a**) and T1-weighed MR image in the venous phase (**b**) show a LEC with mural nodules and enhancing solid component in the ventral wall of the lesion (size of the solid component 5,6 cm). (**c**, **d**) An identical image to a mucinous cystic neoplasm in a different patient. Axial T2 weighted MR image without fat suppression (c) shows a big MCN in the pancreatic body and tail with septations in the dorsal parts. Axial T1-weighted MR image with fat suppression (**d**) shows an enhancing solid component in the dorsal parts of the cystic lesion

**Fig. 6:**
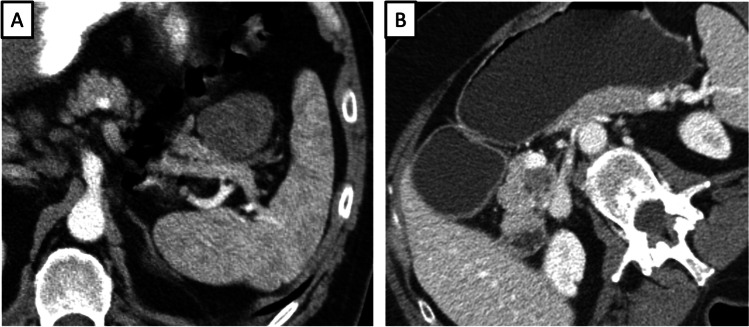
Comparison of lymphoepithelial cyst (LEC) and serous cystic neoplasm (SCN). (**a**) A 47-year-old male patient with LEC in the pancreatic tail, lesion size 4.4 cm. Axial CT image in the arterial phase (**a**) demonstrates the sponge-like appearance of LEC, mimicking SCN. (**b**) A 58-year-old male patient with small SCN in the pancreatic head. Axial CT image in the venous phase (**b**) shows a sponge-like cystic lesion in the dorsal part of the pancreatic head without signs of the infiltration of mesenteric vessels

Overlapping imaging features of LEC and MCN are shown in Fig. 5, and those of LEC and SCN are shown in Fig. 6.

## Discussion

In the present study, which is one of the largest monocentric radiological studies on LEC, we identified the main imaging features of LEC. They can have an imaging appearance as lesions with simple cystic morphology and can be present as complex cystic lesions with either a diffuse subsolid component or a mural nodule. In our case series, the majority of LEC (80%) were visualized as mixed solid-cystic lesions. Our findings correspond to data by Borhani et al. [12], where the authors investigated imaging features of pathologically proven LEC in 10 patients, with 80% being classified as complex cystic lesions. These imaging characteristics can be applied to the diagnostical routine in daily clinical practice. They can potentially help to avoid the imaging misdiagnosis of LEC, which are uncommon pancreatic tumors and show a significant overlap of clinical and imaging features. It is especially important in the cystic lesions that show no typical MCN- and IPMN-imaging features, in such cases in order to avoid unnecessary resection and to increase the accuracy of the diagnosis of an endoscopic or percutaneous biopsy as well as fine needle aspiration (FNA) should be performed.

The first typical clinical feature of LEC is a clear male predominance with a ratio of 9 to 1 (M to F). This finding was the main differential feature of MCNs and is in concordance with two LEC cohorts assembling a male-to-female ratio of 4 to 1 [6] and 7 to 1 [11]. It is reported that the majority of patients with LECs were middle-aged men with an average age of 54 years (range 26–82 years in different studies), which corresponds with our results with the mean age of LEC patients being 50 years. This finding served as the main differential feature of IPMN.

Kim et al. [13] as well as Adsay et al. [6] reported that the most characteristic morphological and imaging finding of LEC is its exophytic location with contour deformation; we were able to verify this finding. This feature can be a result of the development of LEC from epithelial remnants in the lymphatic node that normally has an extrapancreatic location. Adsay [6] and Sewkani [7] reported that LEC could be found in any location of the pancreas including the head, body, and tail. In the following studies [14–18], the LECs were localized in the body and tail, which is in line with our findings with a distribution ratio of the tail to the body of 7:3.

LECs also showed a higher HU attenuation on the unenhanced CT scans when compared to both MCNs and branch-duct IPMNs. This feature can be a result of the granular keratinized material in the cyst fluid intraluminal, one of the pathologic characteristics of the LECs. We found it beneficial in the differentiation of LECs to MCNs and IPMNs. Kudo et al. [19] reported a slight signal reduction in out-of-phase MRI compared to that of in-phase, indicating the co-existence of fat and water. Fukukura et al. [20] stated that LECs could have a lipid component that has negative CT attenuation values. We were not able to verify this finding in our patient collective.

A malignant transformation of LEC has not been reported; epithelial changes are often reactive due to secondary inflammation of the cyst. The elevation of tumor markers is not a distinctive clinical feature of malignancy as around 30–40% of LEC showed elevated CEA and CA 19–9 levels being benign lesions [21].

With imaging, it is possible to diagnose a pseudocyst when a clinical history of acute pancreatitis and follow-up imaging is given. Pseudocysts are most commonly a result of pancreatic inflammation with leakage of the pancreatic duct due to acute pancreatitis. They can also form as a result of pancreatic trauma with parenchymal rupture. They have no malignant potential and normally are followed up or treated with minimal invasive intervention; therefore, we did not include these patients as a control group in our study.

CT and MRI remain the main radiological modalities for the differentiation of cystic pancreatic lesions; however, radiological features of LEC could be indistinguishable from other cystic pancreatic neoplasms. The first diagnostical challenge in the present study was a similar appearance to the branch-duct IPMN. Many authors reported that LECs were not associated with pancreatic duct dilation and were associated with a normal pancreatic duct and pancreatic parenchyma. We have similar findings and found this feature helpful in differentiating the LEC from IPMNs, with IPMNs showing a slight dilation of MPD with a mean value of 4 mm (ranging from 6 to 3 mm). This imaging feature is likely due to the peripheral location of LEC as well.

Secondly, we found radiological appearance indistinguishable from SCNs in some cases, especially from the SCNs that have a subsolid imaging character, which could be found in. In such cases, small cystic components are indistinguishable on CT images showing a sponge-like appearance. Similar features were found in one LEC in our group. In such cases, precise attention should be put to the clinical data of the patient, such as sex and age, as most SCNs are found in older women. Otherwise, both SCNs and LECs are benign lesions and do not require surgery, thus making differentiation between them not clinically relevant. Nevertheless, SCN could be potentially differentiated as they have strong female predominance and occur in elderly patients.

Thirdly, LEC can have a similar appearance to a mucinous cystic neoplasm with oligocystic morphology. The examples above show that preoperative diagnosis of lymphoepithelial cysts from other cystic neoplastic pancreatic lesions, which require surgical intervention, is difficult based solely on radiological criteria. LECs still remain a diagnostic dilemma, and surgical resection is frequently offered because a malignant or premalignant cystic pancreatic lesion cannot be excluded.

Other pancreatic lesions may present as cystic ones or undergo a cystic transformation. Such changes were described by Aldhaheri et al. [22] for undifferentiated carcinoma with osteoclastic giant cells (OGC) of the pancreas that often presents as heterogeneous, solid, and cystic mass with areas of necrosis. Rare tumors or pancreatic metastasis must also be considered as an important differential diagnosis.

The major limitation of our study is the limited number of patients; the explanation for this is the rarity of the LECs. Secondly, there was no standardized imaging protocol for CT and MRI within 12 years because of the retrospective study design. Although some imaging differences between patients were present, the image quality and provided diagnostic information was sufficient for decision-making. Such imaging heterogeneity also reflects realistic clinical workflow. Thirdly, we did not include such lesions with cystic transformation in the comparison analysis and limited it to BD-IPMN, MCN, and CSN.

## Conclusion

In conclusion, our study provides key radiological features of LEC such as the exophytic location most typical in the pancreatic tail and a higher HU attenuation on the unenhanced CT scans. However, the clinical data is still crucial in the differential diagnosis of cystic lesions of the pancreas. The present study showed a strong male predominance of LEC, which is beneficial in the differential diagnosis of MCN and SCN. Compared to branch-duct IPMNs, LECs showed no communication and no dilatation of the main pancreatic duct. The location can also serve as a differential feature with lesions located in the pancreatic head unlikely to be LEC with the most typical location in the pancreatic tail. However, under some circumstances, LECs are indistinguishable from other pancreatic neoplasms, and a correct preoperative diagnosis cannot be done using imaging only. In such cases, a multidisciplinary approach should be strongly considered in order to correlate the imaging finding with clinical data and the possibility of a watch-and-wait approach.

## Data Availability

The datasets generated during and/or analysed during the current study are not publicly available due to sensitive personal information but are available from the corresponding author on reasonable request.
